# AGING, HEALTH, AND CAREGIVING IN ARAB AND ARAB IMMIGRANT POPULATIONS

**DOI:** 10.1093/geroni/igae098.0151

**Published:** 2024-12-31

**Authors:** Carlos Mendes de Leon, Carlos Mendes de Leon

**Affiliations:** Chair; Discussant

## Abstract

There has been a rapid increase in the number of older adults in Arab countries and among Arab immigrants in the US, leading to growing attention to the health and associated care needs due to impairments in physical and cognitive function. Although traditionally, the expectation may have been that the care for these needs would be provided by next of kin, family care resources are becoming less available due to significant social change, such as migration of offspring generations to larger urban centers or other countries. Because formal sources of care are often insufficient or lacking, older Arab adults face an increasingly challenging care environments for their basic care needs. The presentations in this symposium will reflect on aging and caregiving drawing from four geographic sources of information: Egypt, Lebanon, Qatar, and Detroit, Michigan. The Egyptian data come from a pilot study testing the feasibility of conducting a population-based longitudinal study of older adults based on the HRS Around the World Framework, documenting initial findings regarding cognitive impairment. The Lebanon study provides data from a new population-based survey study on caregiving burden and its correlates among older Lebanese adults that provide care to others. The Qatar data come from a qualitative study describing the Muslim religion-inspired belief systems that shape caregiving expectations and practices. The Michigan study presents findings from a culturally adapted intervention for caregivers of older Arab American patients with dementia. Together, these data provide an overview of caregiving needs and practices in Arab and Arab-American populations.

